# Methyl (2*Z*)-2-({*N*-[2-(hy­droxy­meth­yl)phen­yl]-4-methyl­benzene­sulfonamido}­meth­yl)-3-phenyl­prop-2-enoate

**DOI:** 10.1107/S1600536812000864

**Published:** 2012-01-14

**Authors:** R. Madhanraj, S. Murugavel, D. Kannan, M. Bakthadoss

**Affiliations:** a Department of Physics, Ranipettai Engineering College, Thenkadapathangal, Walaja 632 513, India; b Department of Physics, Thanthai Periyar Government Institute of Technology, Vellore 632 002, India; c Department of Organic Chemistry, University of Madras, Maraimalai Campus, Chennai 600 025, India

## Abstract

In the title compound, C_25_H_25_NO_5_S, the O atom of the hy­droxy group is disordered over two positions, with occupancies of 0.820 (2) and 0.180 (2). The sulfonyl-bound benzene ring forms dihedral angles of 31.8 (1) and 60.7 (1)°, respectively, with the hy­droxy­methyl­benzene and phenyl rings. The mol­ecular conformation is stabilized by an intra­molecular O—H⋯O hydrogen bond, generating an *S*(8) ring motif. The crystal packing is stabilized by inter­molecular C—H⋯O hydrogen bonds and C—H⋯π inter­actions.

## Related literature

For background to the pharmacological uses of sulfonamides, see: Korolkovas (1988 ▶); Mandell & Sande (1992 ▶). For resonance effects of acrylate, see: Merlino (1971 ▶); Varghese *et al.* (1986 ▶). For related structures, see: Madhanraj *et al.* (2011 ▶); Aziz-ur-Rehman *et al.* (2010 ▶). For hydrogen-bond motifs, see: Bernstein *et al.* (1995 ▶).
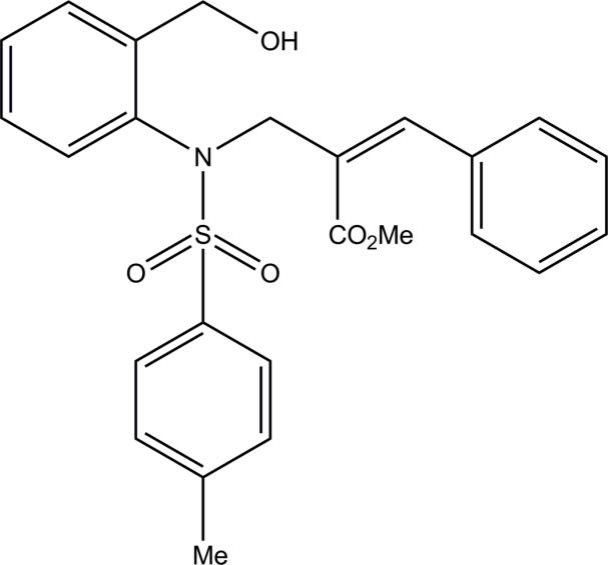


## Experimental

### 

#### Crystal data

C_25_H_25_NO_5_S
*M* *_r_* = 451.52
Triclinic, 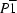

*a* = 7.9528 (3) Å
*b* = 9.5396 (3) Å
*c* = 15.3299 (5) Å
α = 88.253 (2)°
β = 83.571 (1)°
γ = 76.215 (2)°
*V* = 1122.42 (7) Å^3^
*Z* = 2
Mo *K*α radiation
μ = 0.18 mm^−1^
*T* = 293 K
0.23 × 0.21 × 0.16 mm

#### Data collection

Bruker APEXII CCD diffractometer
Absorption correction: multi-scan (*SADABS*; Sheldrick, 1996 ▶) *T*_min_ = 0.959, *T*_max_ = 0.971
25861 measured reflections
7132 independent reflections
5255 reflections with *I* > 2σ(*I*)
*R*_int_ = 0.027

#### Refinement

*R*[*F* ^2^ > 2σ(*F* ^2^)] = 0.042
*wR*(*F* ^2^) = 0.127
*S* = 1.05
7132 reflections
297 parameters
2 restraints
H-atom parameters constrained
Δρ_max_ = 0.31 e Å^−3^
Δρ_min_ = −0.31 e Å^−3^

### 

Data collection: *APEX2* (Bruker, 2004 ▶); cell refinement: *APEX2* and *SAINT* (Bruker, 2004 ▶); data reduction: *SAINT* and *XPREP* (Bruker, 2004 ▶); program(s) used to solve structure: *SHELXS97* (Sheldrick, 2008 ▶); program(s) used to refine structure: *SHELXL97* (Sheldrick, 2008 ▶); molecular graphics: *ORTEP-3* (Farrugia (1997 ▶); software used to prepare material for publication: *SHELXL97* and *PLATON* (Spek, 2009 ▶).

## Supplementary Material

Crystal structure: contains datablock(s) global, I. DOI: 10.1107/S1600536812000864/bt5779sup1.cif

Structure factors: contains datablock(s) I. DOI: 10.1107/S1600536812000864/bt5779Isup2.hkl

Supplementary material file. DOI: 10.1107/S1600536812000864/bt5779Isup3.cml

Additional supplementary materials:  crystallographic information; 3D view; checkCIF report

## Figures and Tables

**Table 1:** Hydrogen-bond geometry (Å, °) *Cg*1 and *Cg*2 are the centroids of the C18–C23 and C8–C13 benzene rings, respectively.

*D*—H⋯*A*	*D*—H	H⋯*A*	*D*⋯*A*	*D*—H⋯*A*
O1*A*—H1*A*⋯O2	0.82	2.23	2.958 (2)	147
C9—H9⋯*Cg*1	0.93	2.80	3.545 (2)	138
C5—H5⋯O4^i^	0.93	2.54	3.429 (2)	160
C14—H14*C*⋯O2^ii^	0.96	2.55	3.359 (2)	143
C12—H12⋯*Cg*1^iii^	0.93	2.72	3.506 (2)	143
C20—H20⋯*Cg*2^iv^	0.93	2.92	3.593 (2)	130

Symmetry codes: (i) 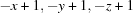; (ii) 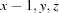; (iii) 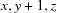; (iv) 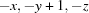.

**Fig. 1.**
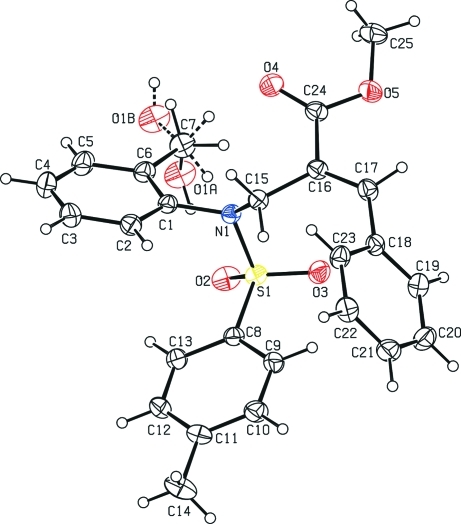
The molecular structure of the title compound, showing 30% probability displacement ellipsoids. H atoms are presented as a small spheres of arbitrary radius. The disordered component is shown.

**Fig. 2.**
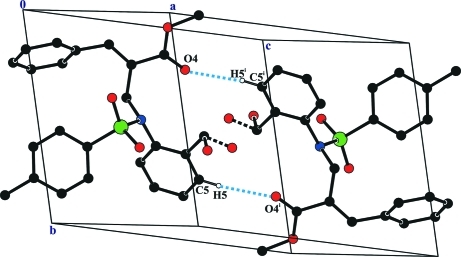
Part of the crystal structure of the title compound showing C—H···O intermolecular hydrogen bonds (dotted lines) generating R22(18) centrosymmetric dimer. [Symmetry code: (i) 1-x, 1-y, 1-z].

**Fig. 3.**
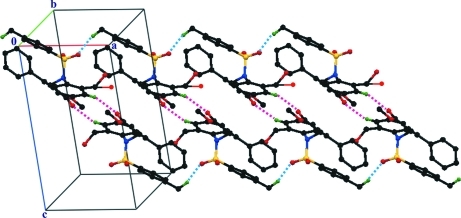
Supramolecular tape formation in (I) whereby centrosymmetrc R22(18) dimeric aggregates sustained by C—H···O (magenta dashed lines) contacts are linked via C—H···O contacts (cyan dashed lines) along [1 0 0].

**Fig. 4.**
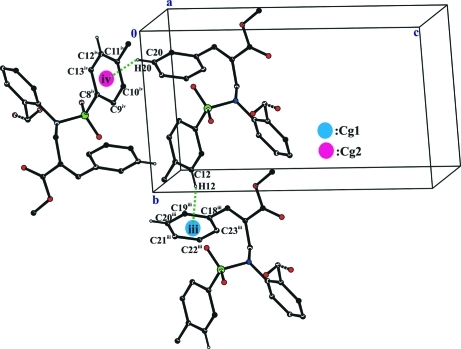
A view of the C—H···π interactions, in the crystal structure of the title compound. Cg1 and Cg2 are the centroids of the (C18–C23) benzene and (C8–C13) benzene rings, respectively. [Symmetry code: (iii) x, 1+y, z; (iv) -x, 1-y, -z.]

## supplementary crystallographic information

### Comment

Sulfonamide drugs are widely used for the treatment of certain infections caused by Gram-positive and Gram-negative microorganisms, some fungi, and certain protozoa (Korolkovas, 1988, Mandell & Sande, 1992). In view of this biological importance, the crystal structure of the title compound has been determined and the results are presented here.

Fig. 1. shows a displacement ellipsoid plot of (I), with the atom numbering scheme. The O1 atom of the hydroxyl group is disordered over two positions with occupancies 0.820 (2):0.180 (2). The S1 atom shows a distorted tetrahedral geometry, with O2—S1—O3[120.0 (1)°] and N1—S1—C8[108.6 (1)°] angles deviating from ideal tetrahedral values. The significant difference in length of the C24—O5 = 1.329 (2) Å and C25—O5 = 1.442 (2) Å bonds is attributed to a partial contribution from the O^-^–C = O^+^–C resonance structure of the O4═C24—O5—C25 group (Merlino, 1971). This feature, commonly observed in the carboxylic ester group of the substituents in various compounds gives average values of 1.340 Å and 1.447 Å respectively for these bonds (Varghese *et al.,* 1986). The sum of bond angles around N1 (346°) indicates that N1 is in *sp*^2^ hybridization. The sulfonyl-bound benzene (C8–C13) ring forms dihedral angles of 31.8 (1)° and 60.7 (1)°, respectively, with the hydroxymethyl benzene (C1–C6) and benzene (C18–C23) rings. The dihedral angle between hydroxymethyl benzene and benzene rings is 41.2 (1)°. The geometric parameters of the title molecule agrees well with those reported for similar structures (Madhanraj *et al.,* 2011, Aziz-ur-Rehman *et al.,* 2010).

The molecule is stabilized by an intramolecular O1A-H1A···O2 hydrogen bond generating an S(8) ring motif (Bernstein *et al.,* 1995) and an intramolecular C-H···π interaction between a sulfonyl–bound benzene H atom and a benzene (C18–C23) ring with a C9—H9···Cg1 seperation of 2.80 Å (Table 1; Cg1 is the centroid of the C18–C23 benzene ring). The crystal packing is stabilized by intermolecular C—H···O hydrogen bonds. The molecules at *x, y, z* and *1-x, 1-y, 1-z* are linked by C5—H5···O4 hydrogen bonds into cyclic centrosymmetric *R_2_^2^*(18) dimers (Fig. 2). These dimers are linked by C14—H14C···O2 hydrogen bonds forming supramolecular tapes running along the [100] directions (Fig. 3). The crystal packing is further stabilized by C—H···π interactions, the first one between a sulfonyl–bound benzene H atom and the benzene ring (C18–C23) of an adjacent molecule, with a C12—H12···Cg1^iii^ seperation of 2.72 Å and the second one between a benzene H atom and the benzene ring (C8–C13) of a neighbouring molecule, with a C20—H20···Cg2^iv^ seperation of 2.92 Å ( Table 1 and Fig. 4; Cg1 and Cg2 are the centroids of the (C18–C23) benzene and (C8–C13) benzene rings, respectively. symmetry code as in Fig. 3).

### Experimental

A solution of *N*-(2-(hydroxymethyl)phenyl)-(4-methylbenzene)sulfonamide (1 mmol, 0.277 g) and potassium carbonate (1.5 mmol, 0.207 g) in acetonitrile solvent was stirred for 15 minutes at room temperature. To this solution, (*z*)-methyl-2-(bromomethyl)-3-phenylprop-2-enoate (1.2 mmol, 0.304 g) was added dropwise till the addition is complete. After the completion of the reaction, as indicated by TLC, acetonitile was evaporated. ETOAc (15 ml) and water (15 ml) were added to the crude mass. The organic layer was dried over anhydrous sodium sulfate. Removal of solvent led to the crude product, which was purified through pad of silica gel (100-200 mesh) using ethylacetate and hexanes (1:9) as solvents. The pure title compound was obtained as a colourless solid (0.435 g, 96% yield). Recrystallization was carried out using ethylacetate as solvent.

### Refinement

Atom O1 is disordered over two positions (O1A/O1B) with refined occpancies of 0.820 (2) and 0.180 (2). The C—O distances of the disordered hydroxyl group were restrained to 1.40 Å. All the H atoms were positioned geometrically, (C–H = 0.93–0.97 Å and O—H = 0.82 Å) constrained to ride on their parent atom, with *U*_iso_(H) =1.5*U*_eq_ for methyl H atoms and 1.2*U*_eq_(C) for other H atoms.

### Crystal data

**Table:**

C_25_H_25_NO_5_S	*Z* = 2
*M**_r_* = 451.52	*F*(000) = 476
Triclinic, *P*1	*D*_x_ = 1.336 Mg m^−^^3^
Hall symbol: -P 1	Mo *K*α radiation, λ = 0.71073 Å
*a* = 7.9528 (3) Å	Cell parameters from 7307 reflections
*b* = 9.5396 (3) Å	θ = 1.3–31.3°
*c* = 15.3299 (5) Å	µ = 0.18 mm^−^^1^
α = 88.253 (2)°	*T* = 293 K
β = 83.571 (1)°	Block, colourless
γ = 76.215 (2)°	0.23 × 0.21 × 0.16 mm
*V* = 1122.42 (7) Å^3^	

### Data collection

**Table:**

Bruker APEXII CCD diffractometer	7132 independent reflections
Radiation source: fine-focus sealed tube	5255 reflections with *I* > 2σ(*I*)
graphite	*R*_int_ = 0.027
Detector resolution: 10.0 pixels mm^-1^	θ_max_ = 31.3°, θ_min_ = 1.3°
ω scans	*h* = −11→11
Absorption correction: multi-scan (*SADABS*; Sheldrick, 1996)	*k* = −13→13
*T*_min_ = 0.959, *T*_max_ = 0.971	*l* = −21→22
25861 measured reflections	

### Refinement

**Table:**

Refinement on *F*^2^	Primary atom site location: structure-invariant direct methods
Least-squares matrix: full	Secondary atom site location: difference Fourier map
*R*[*F*^2^ > 2σ(*F*^2^)] = 0.042	Hydrogen site location: inferred from neighbouring sites
*wR*(*F*^2^) = 0.127	H-atom parameters constrained
*S* = 1.05	*w* = 1/[σ^2^(*F*_o_^2^) + (0.0619*P*)^2^ + 0.1756*P*] where *P* = (*F*_o_^2^ + 2*F*_c_^2^)/3
7132 reflections	(Δ/σ)_max_ < 0.001
297 parameters	Δρ_max_ = 0.31 e Å^−^^3^
2 restraints	Δρ_min_ = −0.31 e Å^−^^3^

### Special details

**Table:**

Geometry. All esds (except the esd in the dihedral angle between two l.s. planes) are estimated using the full covariance matrix. The cell esds are taken into account individually in the estimation of esds in distances, angles and torsion angles; correlations between esds in cell parameters are only used when they are defined by crystal symmetry. An approximate (isotropic) treatment of cell esds is used for estimating esds involving l.s. planes.
Refinement. Refinement of F^2^ against ALL reflections. The weighted R-factor wR and goodness of fit S are based on F^2^, conventional R-factors R are based on F, with F set to zero for negative F^2^. The threshold expression of F^2^ > 2sigma(F^2^) is used only for calculating R-factors(gt) etc. and is not relevant to the choice of reflections for refinement. R-factors based on F^2^ are statistically about twice as large as those based on F, and R- factors based on ALL data will be even larger.

### Fractional atomic coordinates and isotropic or equivalent isotropic displacement parameters (Å^2^)

**Table:**

	*x*	*y*	*z*	*U*_iso_*/*U*_eq_	Occ. (<1)
C1	0.20025 (16)	0.58467 (13)	0.36181 (8)	0.0327 (2)	
C2	0.03176 (18)	0.65951 (14)	0.39145 (9)	0.0401 (3)	
H2	−0.0629	0.6363	0.3699	0.048*	
C3	0.0045 (2)	0.76791 (16)	0.45268 (10)	0.0511 (4)	
H3	−0.1082	0.8175	0.4725	0.061*	
C4	0.1448 (3)	0.80219 (17)	0.48427 (11)	0.0575 (4)	
H4	0.1271	0.8762	0.5248	0.069*	
C5	0.3121 (2)	0.72685 (16)	0.45592 (10)	0.0515 (4)	
H5	0.4056	0.7507	0.4783	0.062*	
C6	0.34408 (18)	0.61594 (14)	0.39460 (9)	0.0393 (3)	
C8	0.04825 (17)	0.61769 (13)	0.16515 (8)	0.0343 (3)	
C9	−0.05349 (19)	0.55105 (14)	0.12048 (9)	0.0410 (3)	
H9	−0.0160	0.4536	0.1064	0.049*	
C10	−0.2116 (2)	0.63118 (16)	0.09707 (10)	0.0469 (3)	
H10	−0.2801	0.5867	0.0672	0.056*	
C11	−0.26927 (19)	0.77612 (15)	0.11741 (10)	0.0443 (3)	
C12	−0.1653 (2)	0.84054 (14)	0.16182 (10)	0.0460 (3)	
H12	−0.2029	0.9380	0.1758	0.055*	
C13	−0.0071 (2)	0.76351 (14)	0.18575 (9)	0.0423 (3)	
H13	0.0615	0.8085	0.2152	0.051*	
C14	−0.4407 (2)	0.8624 (2)	0.09128 (14)	0.0681 (5)	
H14A	−0.4480	0.8451	0.0306	0.102*	
H14B	−0.4493	0.9632	0.0997	0.102*	
H14C	−0.5343	0.8338	0.1268	0.102*	
C15	0.11357 (16)	0.36554 (12)	0.31857 (8)	0.0339 (2)	
H15A	0.0152	0.3908	0.2843	0.041*	
H15B	0.0683	0.3736	0.3801	0.041*	
C16	0.20914 (16)	0.21190 (13)	0.29938 (8)	0.0350 (3)	
C17	0.16244 (17)	0.12583 (14)	0.24467 (9)	0.0393 (3)	
H17	0.2324	0.0328	0.2378	0.047*	
C18	0.01129 (17)	0.16300 (13)	0.19390 (9)	0.0364 (3)	
C19	0.0349 (2)	0.14001 (16)	0.10405 (10)	0.0459 (3)	
H19	0.1452	0.0979	0.0768	0.055*	
C20	−0.1044 (2)	0.17926 (17)	0.05454 (10)	0.0530 (4)	
H20	−0.0870	0.1655	−0.0059	0.064*	
C21	−0.2684 (2)	0.23862 (17)	0.09474 (11)	0.0528 (4)	
H21	−0.3616	0.2661	0.0613	0.063*	
C22	−0.29490 (19)	0.25740 (16)	0.18413 (11)	0.0482 (3)	
H22	−0.4065	0.2948	0.2114	0.058*	
C23	−0.15595 (18)	0.22084 (15)	0.23353 (9)	0.0403 (3)	
H23	−0.1744	0.2350	0.2939	0.048*	
C24	0.36113 (17)	0.15603 (14)	0.34979 (9)	0.0385 (3)	
C25	0.6052 (2)	−0.03467 (18)	0.36773 (12)	0.0552 (4)	
H25A	0.6903	0.0223	0.3604	0.083*	
H25B	0.6568	−0.1303	0.3459	0.083*	
H25C	0.5648	−0.0397	0.4289	0.083*	
N1	0.22546 (13)	0.47007 (10)	0.29838 (7)	0.0317 (2)	
O2	0.36596 (14)	0.61308 (12)	0.18924 (7)	0.0517 (3)	
O3	0.29601 (13)	0.38840 (11)	0.14434 (6)	0.0463 (2)	
O4	0.39010 (15)	0.21719 (12)	0.41165 (8)	0.0565 (3)	
O5	0.46033 (14)	0.03080 (11)	0.31967 (7)	0.0522 (3)	
S1	0.24992 (4)	0.51837 (3)	0.19400 (2)	0.03568 (9)	
C7	0.52803 (19)	0.53288 (18)	0.36886 (11)	0.0509 (4)	
H7A	0.5850	0.5067	0.4217	0.061*	0.820 (2)
H7B	0.5248	0.4441	0.3405	0.061*	0.820 (2)
H7C	0.5370	0.4333	0.3870	0.061*	0.180 (2)
H7D	0.5488	0.5337	0.3053	0.061*	0.180 (2)
O1A	0.62756 (19)	0.60607 (18)	0.31313 (10)	0.0630 (4)	0.820 (2)
H1A	0.5817	0.6274	0.2677	0.095*	0.820 (2)
O1B	0.6587 (8)	0.5806 (8)	0.4002 (4)	0.0630 (4)	0.180 (2)
H1B	0.6916	0.5313	0.4426	0.095*	0.180 (2)

### Atomic displacement parameters (Å^2^)

**Table:**

	*U*^11^	*U*^22^	*U*^33^	*U*^12^	*U*^13^	*U*^23^
C1	0.0352 (6)	0.0318 (5)	0.0329 (6)	−0.0103 (5)	−0.0073 (5)	0.0012 (4)
C2	0.0375 (7)	0.0411 (7)	0.0410 (7)	−0.0077 (5)	−0.0050 (5)	−0.0004 (5)
C3	0.0522 (9)	0.0453 (7)	0.0490 (8)	0.0005 (7)	−0.0006 (7)	−0.0060 (6)
C4	0.0780 (12)	0.0469 (8)	0.0479 (9)	−0.0131 (8)	−0.0075 (8)	−0.0130 (7)
C5	0.0619 (10)	0.0499 (8)	0.0506 (8)	−0.0235 (7)	−0.0166 (7)	−0.0052 (6)
C6	0.0404 (7)	0.0410 (6)	0.0412 (7)	−0.0157 (6)	−0.0117 (5)	0.0007 (5)
C8	0.0372 (6)	0.0345 (6)	0.0325 (6)	−0.0101 (5)	−0.0069 (5)	0.0034 (5)
C9	0.0461 (7)	0.0339 (6)	0.0444 (7)	−0.0090 (5)	−0.0124 (6)	−0.0010 (5)
C10	0.0471 (8)	0.0449 (7)	0.0533 (8)	−0.0144 (6)	−0.0185 (6)	0.0033 (6)
C11	0.0422 (7)	0.0425 (7)	0.0469 (8)	−0.0079 (6)	−0.0077 (6)	0.0134 (6)
C12	0.0571 (9)	0.0318 (6)	0.0468 (8)	−0.0062 (6)	−0.0066 (6)	0.0045 (5)
C13	0.0543 (8)	0.0351 (6)	0.0408 (7)	−0.0139 (6)	−0.0114 (6)	0.0013 (5)
C14	0.0516 (10)	0.0588 (10)	0.0901 (14)	−0.0034 (8)	−0.0194 (9)	0.0235 (9)
C15	0.0310 (6)	0.0329 (6)	0.0398 (6)	−0.0107 (5)	−0.0060 (5)	0.0000 (5)
C16	0.0316 (6)	0.0341 (6)	0.0408 (6)	−0.0093 (5)	−0.0079 (5)	0.0025 (5)
C17	0.0337 (6)	0.0343 (6)	0.0502 (8)	−0.0065 (5)	−0.0083 (5)	−0.0041 (5)
C18	0.0352 (6)	0.0316 (6)	0.0449 (7)	−0.0099 (5)	−0.0096 (5)	−0.0053 (5)
C19	0.0446 (8)	0.0464 (7)	0.0468 (8)	−0.0111 (6)	−0.0021 (6)	−0.0097 (6)
C20	0.0671 (10)	0.0552 (8)	0.0417 (8)	−0.0198 (8)	−0.0144 (7)	−0.0038 (6)
C21	0.0543 (9)	0.0470 (8)	0.0627 (10)	−0.0127 (7)	−0.0288 (8)	0.0006 (7)
C22	0.0341 (7)	0.0471 (7)	0.0645 (9)	−0.0079 (6)	−0.0120 (6)	−0.0089 (7)
C23	0.0368 (7)	0.0434 (7)	0.0430 (7)	−0.0122 (5)	−0.0061 (5)	−0.0076 (5)
C24	0.0355 (6)	0.0372 (6)	0.0448 (7)	−0.0107 (5)	−0.0099 (5)	0.0052 (5)
C25	0.0428 (8)	0.0558 (9)	0.0626 (10)	0.0017 (7)	−0.0180 (7)	0.0089 (7)
N1	0.0315 (5)	0.0334 (5)	0.0332 (5)	−0.0115 (4)	−0.0074 (4)	−0.0002 (4)
O2	0.0446 (6)	0.0671 (7)	0.0514 (6)	−0.0299 (5)	−0.0065 (5)	0.0116 (5)
O3	0.0406 (5)	0.0528 (6)	0.0407 (5)	−0.0009 (4)	−0.0032 (4)	−0.0082 (4)
O4	0.0584 (7)	0.0547 (6)	0.0587 (7)	−0.0063 (5)	−0.0291 (5)	−0.0066 (5)
O5	0.0452 (6)	0.0466 (5)	0.0611 (7)	0.0038 (5)	−0.0211 (5)	−0.0034 (5)
S1	0.03119 (16)	0.04236 (17)	0.03454 (16)	−0.01077 (12)	−0.00391 (11)	0.00105 (12)
C7	0.0365 (7)	0.0594 (9)	0.0609 (9)	−0.0160 (7)	−0.0129 (6)	−0.0015 (7)
O1A	0.0481 (8)	0.0859 (10)	0.0618 (9)	−0.0297 (7)	−0.0067 (6)	0.0066 (8)
O1B	0.0481 (8)	0.0859 (10)	0.0618 (9)	−0.0297 (7)	−0.0067 (6)	0.0066 (8)

### Geometric parameters (Å, °)

**Table:**

C1—C2	1.3926 (18)	C16—C24	1.4897 (17)
C1—C6	1.3966 (17)	C17—C18	1.4717 (18)
C1—N1	1.4484 (15)	C17—H17	0.9300
C2—C3	1.380 (2)	C18—C19	1.3863 (19)
C2—H2	0.9300	C18—C23	1.3900 (19)
C3—C4	1.375 (2)	C19—C20	1.385 (2)
C3—H3	0.9300	C19—H19	0.9300
C4—C5	1.381 (2)	C20—C21	1.375 (2)
C4—H4	0.9300	C20—H20	0.9300
C5—C6	1.395 (2)	C21—C22	1.374 (2)
C5—H5	0.9300	C21—H21	0.9300
C6—C7	1.504 (2)	C22—C23	1.3799 (19)
C8—C9	1.3869 (18)	C22—H22	0.9300
C8—C13	1.3901 (18)	C23—H23	0.9300
C8—S1	1.7535 (13)	C24—O4	1.1998 (17)
C9—C10	1.3859 (19)	C24—O5	1.3288 (16)
C9—H9	0.9300	C25—O5	1.4419 (17)
C10—C11	1.383 (2)	C25—H25A	0.9600
C10—H10	0.9300	C25—H25B	0.9600
C11—C12	1.385 (2)	C25—H25C	0.9600
C11—C14	1.505 (2)	N1—S1	1.6548 (10)
C12—C13	1.379 (2)	O2—S1	1.4323 (10)
C12—H12	0.9300	O3—S1	1.4247 (10)
C13—H13	0.9300	C7—O1B	1.367 (4)
C14—H14A	0.9600	C7—O1A	1.385 (2)
C14—H14B	0.9600	C7—H7A	0.9700
C14—H14C	0.9600	C7—H7B	0.9700
C15—N1	1.4915 (15)	C7—H7C	0.9700
C15—C16	1.5025 (17)	C7—H7D	0.9700
C15—H15A	0.9700	O1A—H7D	1.0542
C15—H15B	0.9700	O1A—H1A	0.8200
C16—C17	1.3315 (18)	O1B—H1B	0.8200
			
C2—C1—C6	120.74 (12)	C20—C19—H19	119.8
C2—C1—N1	119.33 (11)	C18—C19—H19	119.8
C6—C1—N1	119.89 (11)	C21—C20—C19	120.00 (14)
C3—C2—C1	120.35 (13)	C21—C20—H20	120.0
C3—C2—H2	119.8	C19—C20—H20	120.0
C1—C2—H2	119.8	C22—C21—C20	120.15 (14)
C4—C3—C2	119.66 (14)	C22—C21—H21	119.9
C4—C3—H3	120.2	C20—C21—H21	119.9
C2—C3—H3	120.2	C21—C22—C23	120.04 (14)
C3—C4—C5	120.15 (14)	C21—C22—H22	120.0
C3—C4—H4	119.9	C23—C22—H22	120.0
C5—C4—H4	119.9	C22—C23—C18	120.64 (13)
C4—C5—C6	121.63 (15)	C22—C23—H23	119.7
C4—C5—H5	119.2	C18—C23—H23	119.7
C6—C5—H5	119.2	O4—C24—O5	123.50 (12)
C5—C6—C1	117.44 (13)	O4—C24—C16	123.47 (12)
C5—C6—C7	119.57 (13)	O5—C24—C16	113.03 (11)
C1—C6—C7	122.96 (12)	O5—C25—H25A	109.5
C9—C8—C13	120.41 (12)	O5—C25—H25B	109.5
C9—C8—S1	119.85 (10)	H25A—C25—H25B	109.5
C13—C8—S1	119.73 (10)	O5—C25—H25C	109.5
C10—C9—C8	119.29 (12)	H25A—C25—H25C	109.5
C10—C9—H9	120.4	H25B—C25—H25C	109.5
C8—C9—H9	120.4	C1—N1—C15	115.15 (10)
C11—C10—C9	121.12 (13)	C1—N1—S1	115.85 (8)
C11—C10—H10	119.4	C15—N1—S1	115.24 (8)
C9—C10—H10	119.4	C24—O5—C25	116.57 (12)
C10—C11—C12	118.59 (13)	O3—S1—O2	119.99 (7)
C10—C11—C14	120.71 (14)	O3—S1—N1	106.47 (6)
C12—C11—C14	120.69 (14)	O2—S1—N1	105.76 (6)
C13—C12—C11	121.53 (13)	O3—S1—C8	107.61 (6)
C13—C12—H12	119.2	O2—S1—C8	107.99 (6)
C11—C12—H12	119.2	N1—S1—C8	108.60 (6)
C12—C13—C8	119.05 (13)	O1B—C7—O1A	60.6 (3)
C12—C13—H13	120.5	O1B—C7—C6	117.5 (3)
C8—C13—H13	120.5	O1A—C7—C6	114.78 (14)
C11—C14—H14A	109.5	O1B—C7—H7A	49.6
C11—C14—H14B	109.5	O1A—C7—H7A	108.6
H14A—C14—H14B	109.5	C6—C7—H7A	108.6
C11—C14—H14C	109.5	O1B—C7—H7B	132.9
H14A—C14—H14C	109.5	O1A—C7—H7B	108.6
H14B—C14—H14C	109.5	C6—C7—H7B	108.6
N1—C15—C16	112.99 (10)	H7A—C7—H7B	107.5
N1—C15—H15A	109.0	O1B—C7—H7C	108.7
C16—C15—H15A	109.0	O1A—C7—H7C	135.8
N1—C15—H15B	109.0	C6—C7—H7C	108.0
C16—C15—H15B	109.0	H7A—C7—H7C	65.9
H15A—C15—H15B	107.8	H7B—C7—H7C	44.2
C17—C16—C24	120.28 (12)	O1B—C7—H7D	107.1
C17—C16—C15	124.55 (11)	O1A—C7—H7D	49.4
C24—C16—C15	115.12 (11)	C6—C7—H7D	107.9
C16—C17—C18	126.57 (12)	H7A—C7—H7D	143.1
C16—C17—H17	116.7	H7B—C7—H7D	64.9
C18—C17—H17	116.7	H7C—C7—H7D	107.2
C19—C18—C23	118.62 (12)	C7—O1A—H7D	44.3
C19—C18—C17	119.55 (12)	C7—O1A—H1A	109.5
C23—C18—C17	121.83 (12)	H7D—O1A—H1A	71.9
C20—C19—C18	120.50 (14)	C7—O1B—H1B	109.5
			
C6—C1—C2—C3	1.2 (2)	C21—C22—C23—C18	−0.9 (2)
N1—C1—C2—C3	179.20 (12)	C19—C18—C23—C22	−1.3 (2)
C1—C2—C3—C4	0.2 (2)	C17—C18—C23—C22	178.71 (13)
C2—C3—C4—C5	−1.1 (3)	C17—C16—C24—O4	−165.13 (15)
C3—C4—C5—C6	0.6 (3)	C15—C16—C24—O4	12.34 (19)
C4—C5—C6—C1	0.8 (2)	C17—C16—C24—O5	14.26 (19)
C4—C5—C6—C7	−177.31 (15)	C15—C16—C24—O5	−168.28 (11)
C2—C1—C6—C5	−1.67 (19)	C2—C1—N1—C15	−46.81 (15)
N1—C1—C6—C5	−179.64 (12)	C6—C1—N1—C15	131.19 (12)
C2—C1—C6—C7	176.36 (13)	C2—C1—N1—S1	91.87 (12)
N1—C1—C6—C7	−1.61 (19)	C6—C1—N1—S1	−90.13 (12)
C13—C8—C9—C10	−0.4 (2)	C16—C15—N1—C1	−139.33 (11)
S1—C8—C9—C10	−179.19 (11)	C16—C15—N1—S1	81.74 (11)
C8—C9—C10—C11	0.1 (2)	O4—C24—O5—C25	2.6 (2)
C9—C10—C11—C12	0.1 (2)	C16—C24—O5—C25	−176.78 (12)
C9—C10—C11—C14	179.67 (15)	C1—N1—S1—O3	172.29 (8)
C10—C11—C12—C13	0.0 (2)	C15—N1—S1—O3	−49.07 (10)
C14—C11—C12—C13	−179.51 (15)	C1—N1—S1—O2	43.60 (10)
C11—C12—C13—C8	−0.4 (2)	C15—N1—S1—O2	−177.76 (8)
C9—C8—C13—C12	0.6 (2)	C1—N1—S1—C8	−72.09 (10)
S1—C8—C13—C12	179.34 (11)	C15—N1—S1—C8	66.55 (9)
N1—C15—C16—C17	−122.13 (14)	C9—C8—S1—O3	15.29 (13)
N1—C15—C16—C24	60.53 (14)	C13—C8—S1—O3	−163.49 (11)
C24—C16—C17—C18	176.84 (13)	C9—C8—S1—O2	146.17 (11)
C15—C16—C17—C18	−0.4 (2)	C13—C8—S1—O2	−32.61 (13)
C16—C17—C18—C19	127.54 (16)	C9—C8—S1—N1	−99.59 (12)
C16—C17—C18—C23	−52.5 (2)	C13—C8—S1—N1	81.63 (12)
C23—C18—C19—C20	2.5 (2)	C5—C6—C7—O1B	−6.8 (4)
C17—C18—C19—C20	−177.55 (13)	C1—C6—C7—O1B	175.2 (4)
C18—C19—C20—C21	−1.4 (2)	C5—C6—C7—O1A	−75.15 (19)
C19—C20—C21—C22	−0.8 (2)	C1—C6—C7—O1A	106.87 (16)
C20—C21—C22—C23	2.0 (2)		

### Hydrogen-bond geometry (Å, °)

**Table:**

Cg1 and Cg2 are the centroids of the C18–C23 and C8–C13 benzene rings, respectively.

**Table:**

*D*—H···*A*	*D*—H	H···*A*	*D*···*A*	*D*—H···*A*
O1A—H1A···O2	0.82	2.23	2.958 (2)	147.
C9—H9···Cg1	0.93	2.80	3.545 (2)	138.
C5—H5···O4^i^	0.93	2.54	3.429 (2)	160.
C14—H14C···O2^ii^	0.96	2.55	3.359 (2)	143.
C12—H12···Cg1^iii^	0.93	2.72	3.506 (2)	143.
C20—H20···Cg2^iv^	0.93	2.92	3.593 (2)	130.

Symmetry codes: (i) −*x*+1, −*y*+1, −*z*+1; (ii) *x*−1, *y*, *z*; (iii) *x*, *y*+1, *z*; (iv) −*x*, −*y*+1, −*z*.
